# Noninvasive spinal stimulation improves walking in chronic stroke survivors: a proof-of-concept case series

**DOI:** 10.1186/s12938-024-01231-1

**Published:** 2024-04-01

**Authors:** Yaejin Moon, Chen Yang, Nicole C. Veit, Kelly A. McKenzie, Jay Kim, Shreya Aalla, Lindsey Yingling, Kristine Buchler, Jasmine Hunt, Sophia Jenz, Sung Yul Shin, Ameen Kishta, V. Reggie Edgerton, Yury P. Gerasimenko, Elliot J. Roth, Richard L. Lieber, Arun Jayaraman

**Affiliations:** 1 Shirley Ryan AbilityLab, 355 E. Erie St, Chicago, IL 60611 USA; 2 Feinberg School of Medicine, Northwestern University, Chicago, IL 60611 USA; 3 Department of Exercise Science, Syracuse University, Syracuse, NY 13057 USA; 4 Biomedical Engineering Department, McCormick School of Engineering, Northwestern University, Evanston, IL 60208 USA; 5 Rancho Los Amigos National Rehabilitation Center, Broccoli Impossible-to-Possible Lab, Rancho Research Institute, Downy, CA 90242 USA; 6 Neurorestoration Center, Keck School of Medicine, University of Southern California, Los Angeles, CA 90033 USA; 7 Kentucky Spinal Cord Injury Research Center, University of Louisville, Louisville, KY 40202 USA; 8 Pavlov Institute of Physiology, St. Petersburg, Russia; 9 Hines VA Medical Center, Maywood, IL 60141 USA

**Keywords:** Neuromodulation, Stroke, Spinal cord stimulation, Gait training, Rehabilitation

## Abstract

**Background:**

After stroke, restoring safe, independent, and efficient walking is a top rehabilitation priority. However, in nearly 70% of stroke survivors asymmetrical walking patterns and reduced walking speed persist. This case series study aims to investigate the effectiveness of transcutaneous spinal cord stimulation (tSCS) in enhancing walking ability of persons with chronic stroke.

**Methods:**

Eight participants with hemiparesis after a single, chronic stroke were enrolled. Each participant was assigned to either the Stim group (*N* = 4, gait training + tSCS) or Control group (*N* = 4, gait training alone). Each participant in the Stim group was matched to a participant in the Control group based on age, time since stroke, and self-selected gait speed. For the Stim group, tSCS was delivered during gait training via electrodes placed on the skin between the spinous processes of C5–C6, T11–T12, and L1–L2. Both groups received 24 sessions of gait training over 8 weeks with a physical therapist providing verbal cueing for improved gait symmetry. Gait speed (measured from 10 m walk test), endurance (measured from 6 min walk test), spatiotemporal gait symmetries (step length and swing time), as well as the neurophysiological outcomes (muscle synergy, resting motor thresholds via spinal motor evoked responses) were collected without tSCS at baseline, completion, and 3 month follow-up.

**Results:**

All four Stim participants sustained spatiotemporal symmetry improvements at the 3 month follow-up (step length: 17.7%, swing time: 10.1%) compared to the Control group (step length: 1.1%, swing time 3.6%). Additionally, 3 of 4 Stim participants showed increased number of muscle synergies and/or lowered resting motor thresholds compared to the Control group.

**Conclusions:**

This study provides promising preliminary evidence that using tSCS as a therapeutic catalyst to gait training may increase the efficacy of gait rehabilitation in individuals with chronic stroke.

*Trial registration* NCT03714282 (clinicaltrials.gov), registration date: 2018-10-18.

**Supplementary Information:**

The online version contains supplementary material available at 10.1186/s12938-024-01231-1.

## Background

Stroke is the leading cause of adult-onset disability [1]. Despite many advances in gait research in the last decade, about 35% of stroke survivors fail to regain independence in performing activities of daily living due to the impaired function of their affected leg, and about 70% have gait deficits, including reduced walking speeds, asymmetrical walking patterns, and motor coordination issues [2–4].

Walking deficits after stroke mostly derive from a disruption of the corticospinal pathways that play an important role in transmitting sensory–motor commands [5, 6]. To address this, most interventions using non-invasive electrical pulses focus on stimulation of the motor cortex to activate dormant or new pathways [2, 7, 8]. However, while supra-spinal regions can facilitate fine locomotor control, spinal networks ultimately generate the basic locomotor pattern [9, 10]. More interestingly, a recent study using functional MRI showed increased blood-oxygen-level dependent activities in motor cortex following transcutaneous spinal cord stimulation (tSCS) in individuals with stroke [11]. Therefore, we hypothesized that tSCS would facilitate an improvement of gait after stroke. Our previous work, in collaboration with additional researchers, established anatomical and physiological changes in the spinal cord after stroke [12, 13], offering a theoretical basis for testing our hypothesis of targeting the spinal circuits for post-stroke recovery.

Recently, Moshonkina et al. reported functional improvements in post-stroke individuals after 2 weeks of tSCS with standard physical therapy, achieving the minimum clinical important differences (MCID) in the 6 min walk test and comfortable walking speed [14]. The same investigators reported immediate improvements in walking kinematics after a single tSCS session [15, 16]. Notably, however, none of the studies mentioned above investigated the effects of more than 4 weeks of training nor tried to explore the potential neurophysiological differences accompanied with gait outcomes. Consequently, it remains unclear whether tSCS can exert a lasting impact on restoration of function following a stroke.

We investigated whether tSCS combined with symmetry-focused gait training has a sustained effect on gait recovery after chronic stroke. We hypothesized that longer-term gait training (24 sessions) with tSCS would lead to greater sustained improvements in walking function compared to control treatment focused solely on gait training. Specifically, we focused on gait symmetry since such improvements can have lasting effects on balance and overall mobility of stroke survivors [6]. We also expected that gait improvements would be associated with physiological changes in muscle coordination measured from electromyography (EMG) of the paretic side, and spinal excitability determined by the spinal motor evoked responses (sMERs).

## Results

### Spatiotemporal symmetry

After 24 sessions of training, all four stroke participants that received stimulation (Stim group) improved step length symmetry at post training (Post) compared to before training (Pre) (20.0 increase in absolute symmetry index; 95% Confidence Intervals (CIs): [3.6–36.3]; *P* = 0.05). In contrast, in the control group, only Control 3 showed improved step length symmetry (33% increase), which was lower than that of the matched Stim participant (Stim 3: 64% increase). For swing time symmetry, all Stim participants except Stim 3 showed improvements (9.9 increase; 95% CIs [3.5–17.1]; *P* = 0.05), while all controls showed no changes in swing time symmetry (−0.1 change; 95% CIs [−0.8–0.7]; *P* = 0.39).

At the 3-month follow-up (3FU) assessment, all Stim participants continued to demonstrate improved step length symmetry (17.7 increase; 95% CIs [2.3–33.1]; *P* = 0.05) and swing time symmetry (10.1 increase; 95% CIs [5.8–14.5]; *P* = 0.04) compared to before training. The symmetry from Post to 3FU did not change significantly for the Stim participants (step length symmetry: −2.2 decrease, 95% CIs [−4.8–0.7], *P* = 0.10; swing time symmetry: 0.2 increase, 95% CIs [−8.1–8.0], *P* = 0.46), nor the Control participants (step length symmetry: 0.9 increase, 95% CIs [−0.7–2.6], *P* = 0.22; swing time symmetry: 3.7 increase, 95% CIs [1.9–6.5], *P* = 0.15), Furthermore, all four Stim participants exhibited a greater degree of improvement at the follow-up assessment relative to their Pre, surpassing the level of increases observed in the matched Control participants (Table 1). The Control group did not show significant Pre-3FU changes (step length symmetry: 1.1%; 95% CIs [−7.4–12.8]; *P* = 0.45; swing time symmetry*:* 3.6%; 95% CIs [1.4–6.9]; *P* = 0.16). Step length and swing time symmetries of each participant at Pre, Post, and 3FU are reported in Tables 1, 2.

**Table 1:** Step length symmetry of each participant at each assessment and their Pre to Post, Pre to 3FU changes

Participant	Pre	Post	3FU	∆ Post–Pre		∆ 3FU–Pre		∆ 3FU–Post	
				Raw change	% change	Raw change	% change	Raw change	% change
Match 1									
Stim1	53.2	92.6	92.1	39.5	74%	38.9	73%	− 0.5	− 1%
Control1	88.6	80.9	84.2	− 7.7	− 9%	− 4.4	− 5%	3.3	4%
Match 2									
Stim2	90.8	94.9	96.0	4.2	5%	5.3	6%	1.1	1%
Control2	81	70.8	71.0	− 10.2	− 13%	− 10.0	− 12%	0.2	0%
Match 3									
Stim3	51.4	84.5	78.8	33.1	64%	27.4	53%	− 5.7	− 7%
Control3	52.2	69.3	70.7	17.2	33%	18.5	35%	1.4	2%
Match 4									
Stim4	93.5	96.6	92.8	3	3%	− 0.7	− 1%	− 3.8	− 4%
Control4	81.5	83.2	81.8	1.6	2%	0.3	0%	− 1.4	− 2%
Mean (SD)									
Stim	72.2 (23.0)	92.2 (5.4)	89.9 (7.6)	20.0 (19.1)	37% (38)	17.7 (18.6)	33% (36)	− 2.2 (3.1)	− 3% (4)
Control	75.8 (16.1)	76.1 (7.0)	76.9 (7.1)	0.2 (12.4)	3% (21)	1.1 (12.3)	5% (21)	0.9 (2.0)	1% (2)

*Note:* Perfect gait symmetry = 100. Higher value indicates improved symmetry. *3FU* 3-month follow-up

**Table 2:** Swing time symmetry of each participant at each assessment and their Pre to Post, Pre to 3FU changes

Participant	Pre	Post	3FU	∆ Post–Pre		∆ 3FU–Pre		∆ 3FU–Post	
				Raw change	% change	Raw change	% change	Raw change	% change
Match 1									
Stim1	55.5	67.5	61.2	12	22%	5.6	10%	− 6.3	− 9%
Control1	59.3	59.1	60.6	− 0.2	0%	1.3	2%	1.5	3%
Match 2									
Stim2	55.3	76.1	67.3	20.7	37%	12.0	22%	− 8.8	− 12%
Control2	51.7	51.8	54.6	0.1	0%	2.9	6%	2.8	5%
Match 3									
Stim3	54.4	55.3	60.4	0.8	1%	6.0	11%	5.1	9%
Control3	51	49.7	52.5	− 1.3	− 3%	1.4	3%	2.8	6%
Match 4									
Stim4	66.3	72.4	83.2	6.1	9%	16.9	26%	10.8	15%
Control4	69.1	70.1	77.9	1.1	2%	8.8	13%	7.8	11%
Mean (SD)									
Stim	57.9 (5.6)	67.8 (9.1)	68.0 (10.6)	9.9 (8.5)	17% (16)	10.1 (5.4)	17% (8)	0.2 (9.3)	1% (13%)
Control	57.8 (8.4)	57.7 (9.2)	61.4 (11.5)	− 0.1 (1.0)	0% (2)	3.6 (3.5)	6% (5)	3.7 (2.8)	6% (4)

*Note:* Perfect gait symmetry = 100. Higher value indicates improved symmetry. *3FU* 3-month follow-up

### Gait speed

At Post, all Stim participants increased their fast-walking speed (0.33 ± 0.21 m/s increase), which exceeded the MCID of 0.14 m/s. In contrast, only Control 1 met the MCID (0.14 m/s increase) but as a group, the control group did not meet the MCID (0.05 ± 0.08 m/s increase).

At 3FU, two participants (Stim 2, 3) from the Stim group maintained their fast-walking speed over MCID when compared to Pre (Stim 2: 0.14 m/s increase; Stim 3: 0.19 m/s increase), while one Control participant managed to maintain such an improvement (Control 4: 0.22 m/s increase). From Post to 3FU, three Stim participants (Stim 1, 3, and 4) decreased their speed beyond the MCID, with Stim 1 and 4 returning to baseline speed and Stim 3 maintaining a fast-walking speed over the MCID. Table 3 shows the measured speeds and corresponding percent changes from Pre to Post and 3FU, and Post to 3FU for all participants.

**Table 3:** Fast gait speed (m/s) of each participant at each assessment and their Pre to Post, Pre to 3FU changes

Participant	Pre	Post	3FU	∆ Post–Pre		∆ 3FU–Pre		∆ 3FU–Post	
				Raw change	% change	Raw change	% change	Raw change	% change
Match 1									
Stim1	0.66	1.25	0.65	^a^0.59	89%	− 0.01	− 2%	^a^**− **0.60	− 48%
Control1	0.67	0.81	0.75	^a^0.14	21%	0.08	12%	− 0.06	− 7%
Match 2									
Stim2	0.47	0.62	0.61	^a^0.15	32%	^a^0.14	30%	− 0.01	− 2%
Control2	0.52	0.46	0.50	− 0.06	− 12%	− 0.02	− 4%	0.04	9%
Match 3									
Stim3	1.07	1.46	1.26	^a^0.39	36%	^a^0.19	18%	^a^**− **0.20	− 14%
Control3	0.87	0.93	0.89	0.06	7%	0.02	2%	− 0.04	− 4%
Match 4									
Stim4	1.05	1.23	1.04	^a^0.18	17%	**− **0.01	**− **1%	^a^**− **0.19	− 15%
Control4	1.58	1.62	1.80	0.05	3%	^a^0.22	14%	^a^0.18	11%
Mean (SD)									
Stim	0.81 (0.30)	1.14 (0.36)	0.89 (0.31)	0.33 (0.21)	44% (31)	0.08 (0.10)	11% (16)	− 0.25 (0.25)	− 20% (20)
Control	0.91 (0.47)	0.96 (0.49)	0.99 (0.57)	0.05 (0.08)	5% (14)	0.08 (0.11)	6% (8)	0.03 (0.11)	2% (9)

^a^Indicates changes over minimal clinically important difference for gait speed (MCID = 0.14 m/s). 3FU = 3-month follow-up

### 6-min walk test (6MWT)

All Stim participants increased their 6MWT distance (61.6 ± 42.8 m increase) at Post over the MCID of 34.4 m. In contrast, only Control 2 had improvements over the MCID (43.7 m increase), with the matched Stim participant experiencing greater improvements (Stim 2: 49.3 m increase). The Control group had an average improvement of 25.8 ± 13.3 m.

At 3FU, only one participant from each group maintained a walking distance over the MCID compared to before training (Stim 1: 45.0 m increase, Control 4: 58.1 m increase). All participants, except Control 4, decreased their walking distance from Post to 3FU. Table 4 shows the raw data and percent changes from Pre to Post and 3FU, and Post to 3FU for each participant.

**Table 4:** Six-minute walk test distance (m) of each participant at each assessment and their Pre to Post, Pre to 3FU changes

Participant	Pre	Post	3FU	∆ Post–Pre		∆ 3FU–Pre		∆ 3FU–Post	
				Raw change	% change	Raw change	% change	Raw change	% change
Match 1									
Stim1	141.1	266.2	186.1	^a^125.1	89%	^a^45.0	32%	^a^− 80.1	− 30%
Control1	203.3	215.3	192.7	11.9	6%	− 10.6	− 5%	− 22.6	− 10%
Match 2									
Stim2	144.9	194.2	177.0	^a^49.3	34%	32.1	22%	− 17.2	− 9%
Control2	81.1	124.8	113.3	^a^43.7	54%	32.2	40%	− 11.5	− 9%
Match 3									
Stim3	299.0	334.5	276.2	^a^35.5	12%	**− **22.8	− 8%	^a^− 58.3	− 17%
Control3	262.4	283.8	253.0	21.4	8%	− 9.4	− 4%	− 30.8	− 11%
Match 4									
Stim4	235.3	271.6	231.1	^a^36.4	15%	− 4.2	− 2%	^a^−40.5	− 15%
Control4	437.9	464.1	496.1	26.1	6%	^a^58.1	13%	32.0	7%
Mean (SD)									
Stim	205.1 (76.3)	266.6 (57.4)	217.6 (45.7)	61.6 (42.8)	38% (36)	12.5 (31.4)	11% (19)	^a^− 49.0 (26.7)	− 8% (9)
Control	246.2 (148.4)	272.0 (143.7)	263.8 (165.1)	25.8 (13.3)	19% (24)	17.6 (33.6)	11% (21)	− 8.2 (28.0)	− 6% (9)

^a^Indicates changes over minimal clinically important difference for 6MWT (MCID = 34.4 m). *6MWT* 6 min walk test. *3FU* 3-month follow-up

### Muscle synergy analysis during walking

**Fig. 1:**
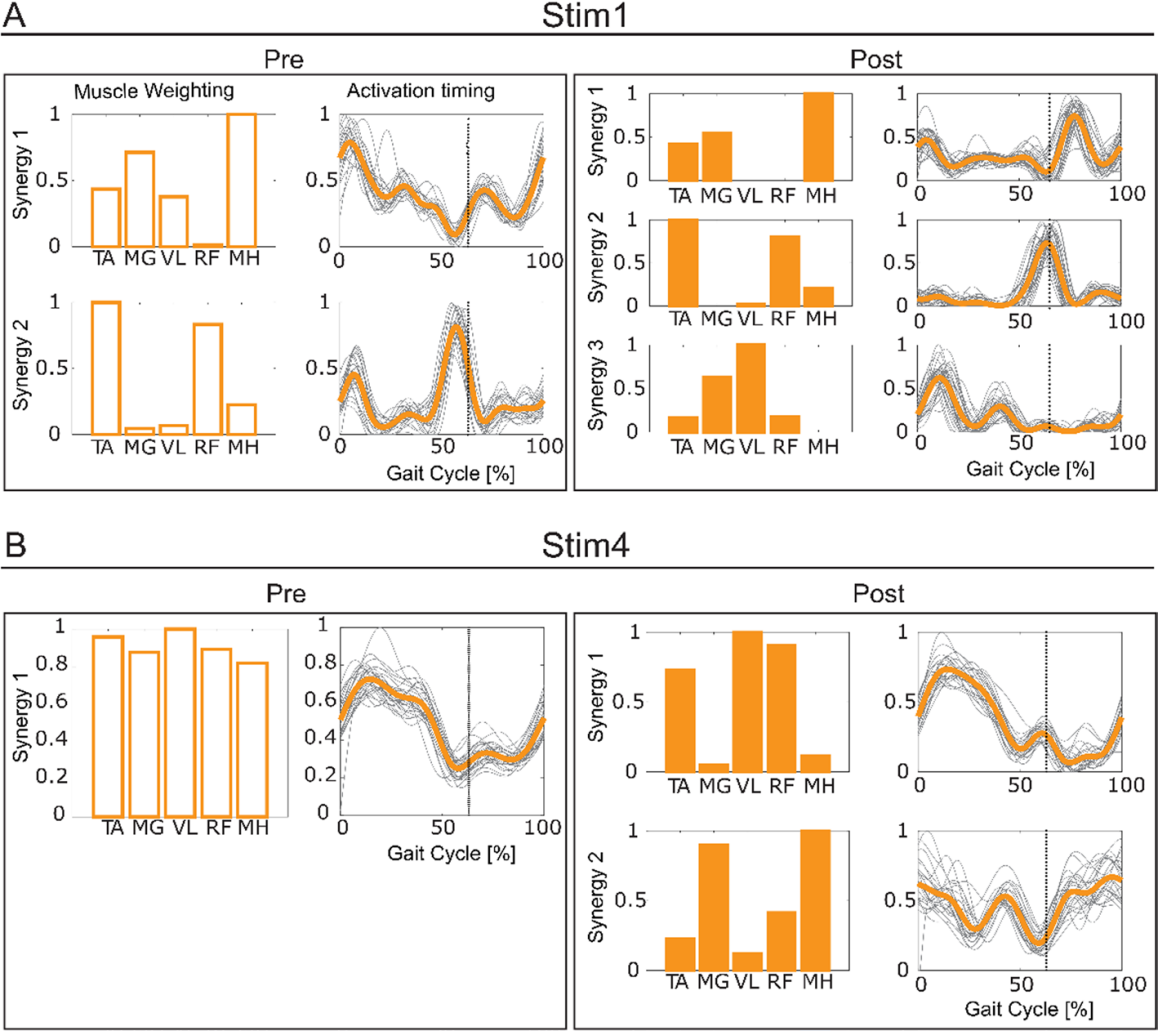
Muscle synergy analysis results. Muscle synergy weightings and synergy activation pattern profiles of the Stim participants with increased number of muscle synergies Post-intervention (**A**: Stim 1, **B**: Stim 4). Vertical dashed line indicates toe off timing. *TA* tibialis anterior, *MG* medial gastrocnemius, *VL* vastus lateralis, *RF* rectus femoris, *MH* medial hamstring

Muscle synergies indicate synchronous neural commands to execute each phase of gait cycle and the group of muscles that are activated together in response to a neural command [17]. The number of muscle synergies measured from electromyography (EMG) in the paretic side was compared between Pre and Post assessments. Two Stim participants (Stim 1–4) increased the number of synergies after the intervention, which has been considered as an indication of improved neuromuscular coordination after stroke [18]. None of the Control participants showed an increase. For Stim 1, the number of synergies increased from two to three (Fig. 1A). Specifically, at Post, an additional synergy was observed with a dominant activity in vastus lateralis (VL) during loading phase (synergy 3 in Fig. 1A Post). For Stim 4, a single synergy was observed at the baseline with a strong response at stance phase (Fig. 1B Pre). At Post, an extra synergy was present in late stance and swing phase with a dominant activation at medial hamstring (MH) and medial gastrocnemius (MG) muscles (synergy 2 in Fig. 1B Post).

### Resting motor threshold (RMT)

**Fig. 2:**
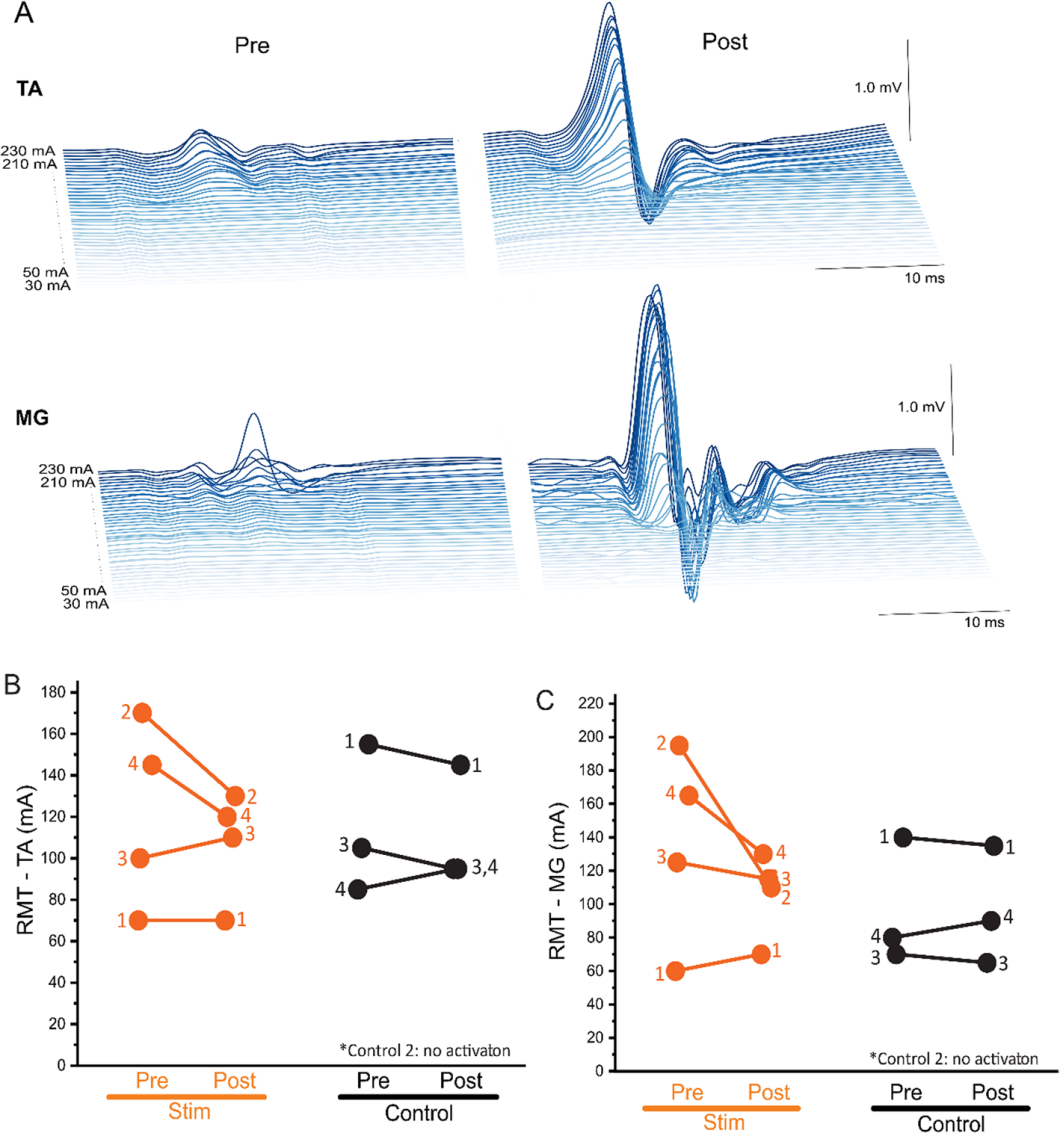
Spinal motor evoked responses (sMERs) results. **A** The responses recorded at Pre and Post-intervention of Stim 2 in the TA and MG muscles of the paretic leg at stimulation intensities at L1 ranging from 50 to 180 mA (5 mA increments). Notably, both muscles responded at lower stimulation intensity (i.e., reduced RMT) at Post compared to Pre-intervention indicating improved spinal excitability. **B**, **C** Individual (line graph; identified by ID within groups) Pre–Post RMT changes for **B** TA muscle and **C** MG muscle. *RMT* resting motor threshold, *TA* tibialis anterior, *MG* medial gastrocnemius

Two participants in the Stim group exhibited a decrease in Post-intervention RMTs compared to Pre-intervention in tibialis anterior (TA) (Fig. 2; Stim 2: 24%, Stim 4: 17% decrease) and medial gastrocnemius (MG) (Stim 2: 44%, Stim 4: 21% decrease) (refer to Additional file 1: Table S1). Notably, these participants had the highest RMT levels at baseline. All other participants exhibited minimal Pre–Post changes in RMTs (≤ 10 mA), with no significant changes from Pre to Post on average (Stim TA: −14 mA, 95% CIs [−33–5], *P* = 0.14; Stim MG: −30 mA, 95% CIs [−66–0], *P* = 0.12; Control TA: −3 mA, 95% CIs [−3–3], *P* = 0.17; Control MG: 0 mA, 95% CIs [0–5], *P* = 0.50). Control 2 did not demonstrate any spinal motor evoked responses (sMERs).

### Participants’ self-report

None of the participants experienced self-reported pain or discomfort during or following the protocol. Informal reports on the effects of the intervention from subjects in the Stim group included: “It has helped me with the stairs and engaging my core in a dynamic way”, “Walking around my house without my cane has gotten a lot better” and “My standing and walking without my brace has gotten better”. Stim group subjects also described improvements in proprioception: “I feel my feet,” and “I feel more aware of my leg after starting this.” None of the participants in the control group provided explicit indications of intervention effects.

## Discussion

In this case series study, we present preliminary evidence that tSCS applied during gait training enhances walking ability, demonstrating increases in spatiotemporal gait symmetry and clinically meaningful improvements in gait speed and walking distance after 24 sessions in individuals with chronic stroke. The clinical findings were accompanied by selected neurophysiological changes.

Our study revealed that all participants in the Stim group maintained or improved step length and swing time symmetry at Post training. Additionally, improvements over the MCID were seen for all four Stim participants for both, 6MWT and fast gait speed, at Post training, however, most participants did not retain the improvements from Post to 3FU and went back to baseline level (except Stim 1 in 6MWT, and Stim 2 and 3 in gait speed). This suggests tSCS might boost the initial endurance and speed improvements when combined with gait training but may be reversed to baseline levels if exercise is discontinued. In contrast, at 3FU, the Stim group continued to maintain a better spatiotemporal symmetry compared to Pre. Such positive gait symmetry changes have been linked with an increased quality of life due to better balance, reduced fall risks, and increased independence as individuals could better reintegrate into the community [6, 19]. These observations support the hypothesis that tSCS paired with gait training focused on gait symmetry facilitates improved gait performance and walking patterns following the intervention. However, this comprehensive improvement in gait function disappeared at 3FU, showing only lasting improvement in gait symmetry.

Notably, we observed a variety of patterns for symmetry improvement within the stimulation group. Specifically, Stim 1 and Stim 3 demonstrated improved step length symmetry (74%–64%), while Stim 2 and Stim 4 exhibited improvements in swing time symmetry (37%–9%). These patterns seemed to be linked to the individuals’ baseline gait symmetry, with more substantial improvements observed in metrics with greater deficits. Importantly, the improvements in one aspect of symmetry were not at the sacrifice of other symmetry metrics for any of the Stim participants. This finding highlights the importance of providing individualized training instructions and stimulation parameters to maximize the effect of tSCS in future studies aimed at enhancing activity-dependent learning.

To probe the underlying neuromuscular changes associated to gait performance after the intervention, we conducted muscle synergy analysis to evaluate neural activity during movement and sMERs to explore the changes in excitability at the local spinal networks. In our study, the two Stim participants (Stim 1–4) who demonstrated an increased number of muscle synergies had the greatest improvements of spatiotemporal symmetry at their 3FU, which is aligns to the findings by Clark et al. suggesting the number of muscle synergies is positively correlated with step length symmetry [18]. Additionally, not only the number of synergies increased, but also the structure of the new synergies reflected those found in healthy locomotion. For Stim 1, Synergy 1 at baseline unmerged into two separate synergies: Synergy 1 with dominant activity in VL for early stance and Synergy 3 with dominant activity in MH for early swing, which is consistent on what is found in healthy locomotion [20]. This observation, combined with earlier studies indicating the involvement of local spinal networks in muscle synergies activation [21, 22], indicates that tSCS may fine-tune muscle activation patterns that could lead to sustained improvements in motor behaviors. Hence, we propose that tSCS might be a viable approach for altering muscle synergies in stroke and therefore targeting the underlying gait deficits of this population.

Additionally, in the sMERs test, two Stim participants (Stim 2–4), who had the highest baseline RMTs, exhibited a substantial decrease in RMT Post-intervention whereas all Control participants showed only minimal changes. This decreased RMT suggests an enhancement in the participants’ spinal excitability after training, a finding in line with earlier studies on tSCS [23–26]. These previous studies demonstrated that tSCS appears to prime spared spinal networks and increase net excitability [27–29] allowing supra-spinal and peripheral inputs to exceed the motor thresholds needed to generate voluntary movement [28]. It has been suggested that the stimulation may reorganize the cortico-reticulo-spinal circuits through this convergence between residual supra-spinal commands and activated afferent pathways, potentially accounting for the persistent motor recovery even in the absence of stimulation [25, 30]. However, these findings lack generalizability since the changes were seen only in some Stim participants. Future research is required to elucidate the significance of spinal cord RMTs within the context of stroke pathology.

A larger sample size is warranted to understand how neurophysiological and functional outcomes after tSCS are correlated and how the changes in spinal excitability are linked to stroke recovery. A post hoc calculation, based on our primary outcome of spatiotemporal gait symmetry, indicated that a total of 50 participants (25 in each group) are required to provide 80% power at a two-sided 5% significance level. Furthermore, the tSCS stimulation parameters used were primarily based on therapist observation, potentially contributing to the observed between-subject variability in outcomes at both Post and 3FU. Additionally, while the pairs were matched as best as possible, our pairs were not matched based on our primary outcome measure (symmetry). This underscores the necessity for personalized tSCS, where stimulation parameters can be tailored to address each individual’s specific gait deficit. Notably, Bogacheva et al. (2023) have employed a gait phase-dependent tSCS stimulation protocol in stroke survivors [15]. This protocol alters the stimulation site based on the gait phase, with stimulation at the T12 vertebrae during the swing phase and at the L1 vertebrae during the stance phase. Nonetheless, the study assessed the effect of stimulation in only a single session intervention. For future studies, we could combine our approach—which provides extended gait training combined with tSCS—with the phase-dependent tSCS stimulation protocol to target improvements in individual-specific gait deficits. However, a more in-depth understanding of stimulation parameters is crucial before conducting accurate personalized tSCS interventions. In summary, the promising preliminary results of this study portend a significant effect when the upcoming clinical trial (HD106015, Clinical Trials Number: NCT05167786) is completed (*n* = 50).

## Conclusions

In summary, this pilot case series successfully demonstrates the feasibility and potential benefit of implementing tSCS in combination with symmetry-focused gait training for individuals with chronic stroke. Our findings suggest that further research is warranted to unlock the potential of tSCS as a neuromodulation technique aimed at improving function in individuals post-stroke.

## Methods

### Study design, setting, and participants

This two-arm, unblinded, pilot case series study was conducted from 2018 through 2020 at Shirley Ryan AbilityLab in Chicago, Illinois. The trial protocol was approved by the institutional review board at Northwestern University. All participants provided written informed consent. Northwestern University Institutional Review Board (IRB) approved this study (IRB protocol #00206430). The study design and conduct complied with all relevant regulations regarding the use of human study participants and was conducted in accordance with the criteria set by the Declaration of Helsinki. The trial protocol was preregistered at ClinicalTrials.gov (NCT03714282).

The participants in this study were recruited by convenience sampling from the Shirley Ryan AbilityLab. Participant inclusion criteria included the following: age over 18 years, at least 1 year post-stroke, hemiplegia secondary to a single stroke, Functional Ambulation Category of 2 or greater, able to provide informed consent, not currently receiving physical therapy services, not participating another clinical trial at the time of the intervention and in the months prior to, and physician approval. Participant exclusion criteria were the following: ataxia, multiple stroke history, currently taking a Selective Serotonin Reuptake Inhibitor or Tricyclic Antidepressant, botulinum toxin injection in the lower extremity within the last 4 months, Modified Ashworth Scale of 3 or greater in the lower extremity, pregnancy or nursing, presence of pacemaker, active pressure sores, unhealed bone fractures, peripheral neuropathies, painful musculoskeletal dysfunction due to active injuries or infections, severe contractures, medical illness limiting ability to walk, active urinary tract infection, clinically significant depression, psychiatric disorders or ongoing drug abuse, metal implants in spine, history of cancer or cancer remission < 5 years.

Eight participants were assigned to either the Stim group (*N* = 4, tSCS + gait training) or Control group (*N* = 4, gait training alone). To minimize possible confounding factors, each Stim group participant was matched to a Control participant for age (± 3 years), time since stroke (± 3 years), and self-selected gait speed (± 0.14 m/s, based on MCID [31]) (Table 5). All subjects were able to ambulate without any hand support.

**Table 5:** Participants’ demographics

	Match1		Match2		Match3		Match4	
ID	Control1	Stim1	Control2	Stim2	Control3	Stim3	Control4	Stim4
Sex	Female	Female	Male	Male	Male	Female	Male	Male
Age (years)	48	49	64	67	59	56	63	61
Time since Stroke (years)	8	11	6	5	2	2	6	9
Stroke type	Isch	Isch	Isch	Hemo	Isch	Isch	Hemo	Hemo
Paretic-side	Left	Right	Left	Left	Right	Left	Left	Right
Self-selected Speed(m/s)	0.56	0.57	0.51	0.37	0.75	0.81	0.99	0.85
FAC score	4	4	5	5	5	5	4	5
AFO	None	None	Solid	Articulated	Articulated	Articulated	None	Flexible
Assistive device	None	None	None	None	None	None	None	None

*Note:* *Isch* ischemic, *Hemo* hemorrhagic, *FAC* functional ambulation category, *AFO* ankle–foot orthoses

### Study protocol

Participants completed 45 min of gait training, 3 times per week, for 8 weeks for a total of 24 sessions with a primary focus on improving spatiotemporal gait symmetry. The Stim group received tSCS during gait training while the Control group received gait training only. Gait performance outcomes were assessed at 3 timepoints: before training (Pre), after all training sessions (Post), and in a 3 month follow-up after the last training session (3FU). All gait assessments were completed without tSCS. Neurophysiological measures were also assessed at Pre and Post timepoints to explore the underlying neurophysiological variations contributing to gait performance changes.

### Gait training protocol

**Fig. 3:**
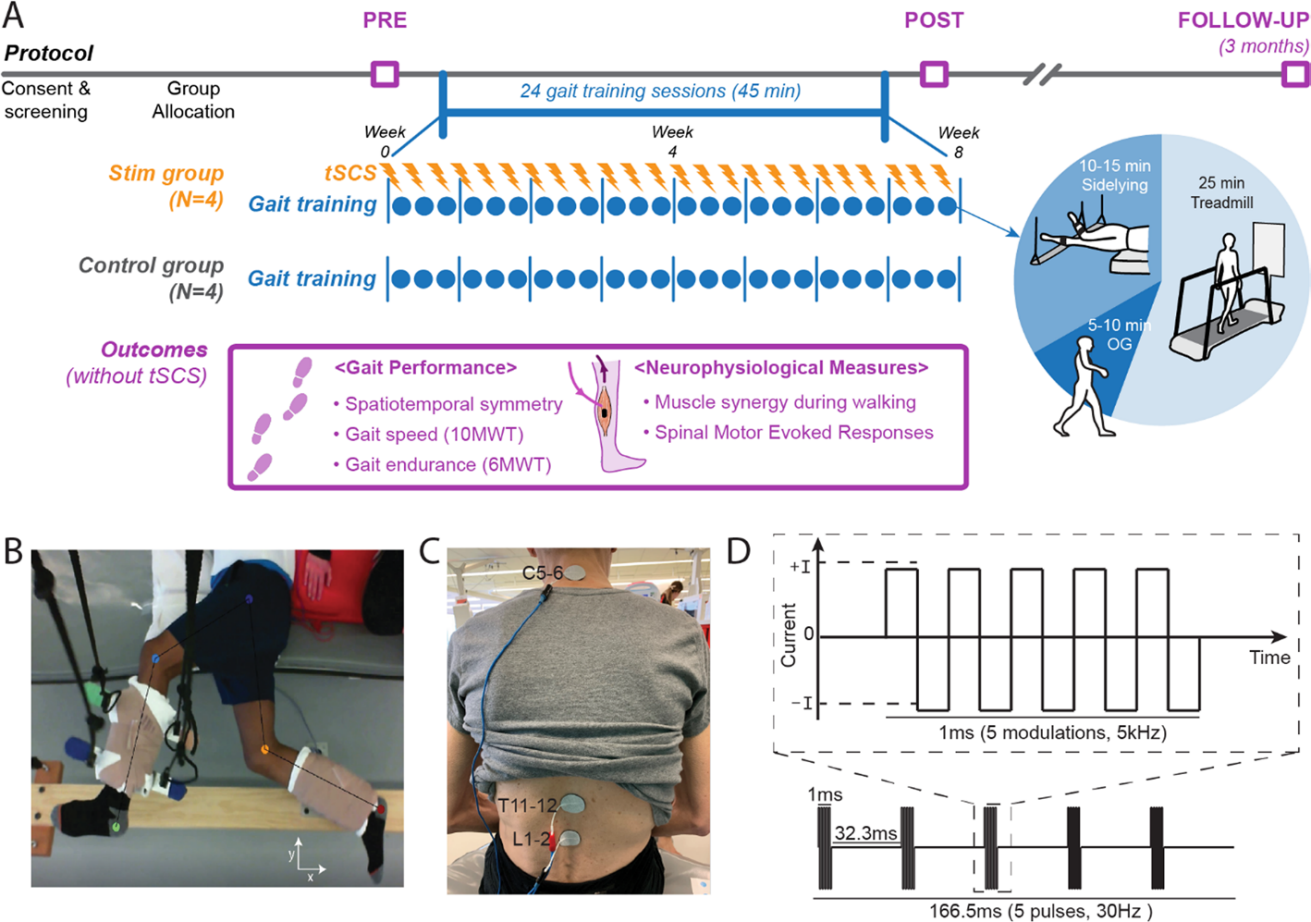
Study protocol and stimulation setup. **A** Overall experimental protocol. **B** Top–down view of position of the legs extended beyond the edge of the table and supported with vertically cables during the side-lying training of a participant (Stim 2). **C** tSCS delivered using surface electrodes on the skin between the C5–6, T11–12, and L1–2 spinous processes (cathode) and a surface electrode on each anterior crest (anode, not shown). **D** Schematic representation of biphasic pulse sequence used for tSCS. *tSCS* transcutaneous spinal cord stimulation, *OG* overground walking, *10MWT* 10-m walk test, *6MWT* 6-min walk test

During training sessions, all participants completed locomotor training in three positions: side-lying (10–15 min), treadmill (25 min), and overground (5–10 min) (Fig. 3A). The side-lying training was intended to train rhythmic and symmetrical lower-limb movements in a gravity neutral position as participants laid on their non-paretic side (Fig. 3B) [32, 33]. Treadmill locomotion was performed on a treadmill (C-Mill^®^, Motek Medical) that provided real-time feedback on spatiotemporal gait parameters. Participants then transitioned to overground locomotion to promote functional carryover. As training sessions progressed, participants spent less time in side-lying training and more time on overground ambulation. All the gait training was conducted with a licensed physical therapist. The physical therapist provided verbal cue for spatiotemporal symmetry in both overground and treadmill trainings. For treadmill sessions, the participants were encouraged not to use the handrail but were allowed to hold it for support if necessary.

### Stimulation settings

A custom-built, constant current, spinal stimulator (BioStim-5, Cosyma, Moscow, Russia) [34] provided tSCS to the Stim group during gait training. tSCS was delivered via cathode electrodes (3.2 cm diameter, ValuTrode, Axelgaard Ltd., Fallbrook, CA, USA) placed on the skin between the C5–6, T11–12, and L1–2 vertebrae (Fig. 3C) [35]. Anode electrodes ( 7.5 × 13 cm, UltraStim, Axelgaard Ltd., Fallbrook, CA, USA) were placed bilaterally on the anterior iliac crests. Stimulation consisted of a continuous, biphasic waveform, cathodic-leading, with rectangular 1-ms pulses (0.5 ms per phase) at 30 Hz, modulated at 5 kHz (Fig. 3D). Subthreshold stimulation intensities (i.e., below the resting motor threshold) were explored since they are known to be superior to suprathreshold stimulation when targeting improved motor activation in a rat model [36]. The motor threshold was determined by the spinal motor evoked responses (sMERs) test (see the details at the outcome assessments section). C5–6 was also stimulated as a prior tSCS study reported the addition of C5–6 with stimulation to T11–12 and L1–2 immediately improved non-voluntary stepping performance in participants without neurologic conditions [23]. The study suggested these sites are consistent with the long propriospinal system modulating the lumbosacral locomotor circuit. For each Stim participant, varying combinations of subthreshold stimulation intensities were assessed during ambulation. For each intensity combination, walking performance was recorded using GAITRite electronic walkway (CIR System Inc., NJ, USA) and through the observational evaluation of physical therapists. The objective was to achieve the most symmetrical gait pattern, as gauged by both GAITRite outcomes (step length and swing time symmetry) and clinical observations. Table 6 lists the intensities of tSCS that were applied at each stimulation location by Participant ID#.

**Table 6:** Stimulation intensities used during gait training

Participant	Stimulation intensity			Stimulation intensity relative to the T11 and L1’s resting motor threshold
	C5 (mA)	T11 (mA)	L1 (mA)	
Stim1	30	34	69	83%
Stim2	10	71	74	46%
Stim3	45	49	51	44%
Stim4	0	119	102	69%

### Intervention adherence and tolerability

To monitor the participants’ safety during the tSCS intervention, we documented informal self-reports on whether there was any discomfort or pain during each training session. We also measured heart rate and blood pressure at the start and end of each session to ensure the values were in the normal range. All subjects tolerated and adhered to the training sessions well, with no reported adverse effects.

## Outcome assessments

### Performance based tests

- *Spatiotemporal gait symmetry.* Participants walked at their self-selected velocity along the 8 m GAITRite electronic walkway (CIR System Inc., NJ, USA) placed in the middle of a 14 m walkway. Each participant completed three trials to account for trial-to-trial variance, and the results were averaged. For each trial, spatiotemporal gait measurements of step length and swing time were extracted. Gait symmetry, the difference between a subject’s paretic (P) and non-paretic (NP) side, was calculated by the following Symmetry Index equation
  $$Symmetry\, Index = \left( {1 - \left| {1 - \frac{NP}{P}} \right|} \right) \times 100\% .$$

This calculation results in a maximum value of 100% irrespective of which limb demonstrates greater values, with improvements observed as positive values [37]. The symmetry indices of three trials for each participant were averaged.

- *Fast gait speed.* To measure gait speed, participants performed the 10 m walk test (10MWT) at fast velocity [38]. The test was repeated over 3 trials and the average speed of the three trials was calculated.
- *6 min walk test (6MWT).* The 6MWT was conducted to examine gait endurance. Participants were instructed to complete 6 min of overground walking, covering as much distance as possible [39].

### Neurophysiological outcomes

- *Electromyography* (*EMG) acquisition during walking.* Surface EMG (Trigno, Delsys, Inc.) was recorded at Pre and Post in five muscles (rectus femoris, RF; vastus lateralis, VL; medial hamstring, MH; tibialis anterior, TA; and medial gastrocnemius, MG) as participants walked at their self-selected speed for 10 m. All EMG data were collected at 2000 Hz. The selected EMG signals from each participant were band-pass filtered from 40 to 500 Hz with a zero-lag fourth-order Butterworth filter, demeaned, rectified, and low-pass filtered with a zero-lag fourth-order Butterworth filter at 4 Hz [18]. To facilitate comparison between subjects, the filtered signal was normalized to its peak value and resampled into 100% of the gait cycle from heel strike to heel strike.
- *Muscle synergy analysis.* The concept of muscle synergies indicates synchronous neural commands to execute each phase of gait cycle and the group of muscles that are activated together in response to a neural command [17]. We conducted non-negative matrix factorization to obtain the EMG-based muscle synergy analysis during walking [20, 40]. Muscle activity during walking can be grouped into sets of co-excited muscles, termed as muscle modules or synergies [41]. Studies have identified well-coordinated gait in healthy individuals can be produced by four or five group of synergies [18, 20, 42]. Recent evidence suggests that disinhibition and/or hyperexcitation of the brainstem descending pathways and intraspinal motor network diffuse spastic synergistic activation post-stroke [43]. As a result, simplified or merged muscle synergies compared to non-impaired individuals are typically observed and has been found to predict their degree of impairment [18]. Furthermore, previous studies suggest that muscle synergies are encoded in the spinal cord [18, 21, 22, 44], therefore we hypothesized that modulating spinal networks with tSCS may lead to positive changes in motor control of stroke survivors that could translate to sustained functional gait changes. Previous research suggested that the increase in number of muscle synergies indicate improvement in neuromodular complexity [18]. To determine the number of muscle synergies necessary to reconstruct the original EMG signal, the variability accounted for (VAF) was calculated and used as the reconstruction quality criterion given by
  $$VAF=\frac{1-{({EMG}_{o}-{EMG}_{r})}^{2}}{{EMG}_{o}^{2}}\ge 90\%,$$

where ${{\text{EMG}}}_{{\text{o}}}$ is the original EMG signals, ${{\text{EMG}}}_{{\text{r}}}$ is the reconstructed EMG signals calculated by multiplying muscle group weightings and activation timing patterns [18]. The number of motor synergies of each walking trial was chosen such that the VAFs exceeded 90% [18].

- *Spinal motor evoked responses* (*sMERs).* Following the methods of our previous work [12], sMERS were performed as participants laid supine and EMG was recorded bilaterally from the same five muscles used for muscle synergy analysis. Surface EMG activity of sMERs was recorded with pairs of bipolar Ag–AgCl surface electrodes (2.5 cm diameter, GS26, Bio-Medical Instruments, Michigan USA). Stimulation was delivered at L1–2 vertebrate site using monophasic, square-wave, single pulses at 5 mA increments, increasing from 5 to 250 mA or until the subject reached maximum tolerance. Each stimulation intensity was delivered three times. EMG signals were sampled at 4000 Hz and band-pass filtered (fourth-order Bessel filter, 30–2000 Hz) by the PowerLab 16/35 data acquisition system operated with LabChart 7.2 Software (AD Instruments, Australia). Resting motor thresholds (RMTs) were calculated for TA and MG as the lowest current intensity at which two out of the three trials had a peak-to-peak amplitude greater than 0.05 mV [45]. TA and MG were chosen since they exhibited primary weakness in all participants on the paretic side based on the manual muscle testing. The RMTs are used as a reference for the current intensity to be used with continuous stimulation during the intervention. A reduction in RMT after intervention may indicate increased motoneuron excitability, implying that the motoneurons are more responsive to supra-spinal input [12, 24].

### Participants’ self-report

- Participants self-reports were obtained at the conclusion of the intervention for the Stim group, supplemented by any comments they provided throughout the 8 week intervention period.

## Data analysis

- ***Bootstrapping for spatiotemporal symmetry and RMTs.*** For gait symmetry, bootstrap methods were performed to statistically verify changes in the outcomes Post and at 3FU relative to Pre, and 3FU relative to Post (SPSS v27.0, IBM, Inc., Chicago, IL). Additionally, bootstrap methods were performed to evaluate the changes in RMTs from Pre to Post. Bootstrapping is a nonparametric statistical analysis that employs resampling techniques and has been effectively used in studies with small sample sizes [47]. Specifically, bootstrapping resamples each original data set with replacement, and recombines it to create bootstrap sets, from which the means and 95% confidence intervals (CIs) were obtained. We constructed 1000 bootstrap samples for each outcome and calculated the Pre–Post, Pre-3FU, and 3FU-Post mean raw symmetry index differences, and the Pre–Post means differences of the RMTs of the resampled data to create statistical results. Then, 95% CIs of the differences were constructed to test the null hypothesis of no difference in the mean. Since we hypothesized that the outcomes would improve in subsequent timepoints, we used a one-tailed paired t-test. The raw changes in spatiotemporal symmetry indexes and RMT changes are presented in the results with 95% CIs and the *P-*value from the bootstrap. The level of significance was set at *P* < 0.05.
- ***Minimum clinical important differences*** (***MCID) for gait speed and 6MWT.*** For gait speed and 6MWT, MCID was used to assess for meaningfulness of improvements (gait speed MCID = 0.14 m/s; 6MWT distance MCID = 34.4 m) [31]. These thresholds are defined as the smallest changes in health-related measures that patients perceive as meaningful improvements in rehabilitation. This approach was chosen over relying solely on statistical significance since a statistically significant change may not always translate into a meaningful improvement in rehabilitation outcomes [48].
- ***Muscle synergies analysis.*** The change in number of muscle synergies was reported in the results.

### Supplementary Information

**Additional file 1: Table S1**. Resting motor threshold (RMT, mA) of sMERs of each participant at each assessment and their Pre to Post changes. *RMT* resting motor threshold, *sMER* spinally motor evoked responses, *TA* tibialis anterior, *MG* medial gastrocnemius.

## Data Availability

The data and code for analysis used in this paper can be made available upon request.

## Abbreviations

tSCS: Transcutaneous spinal cord stimulation
MCID: Minimum clinical important differences
IRB: Institutional Review Board
Isch: Ischemic
Hemo: Hemorrhagic
Pre: Before training
Post: After all the training sessions
3FU: 3-Month follow-up after the last training session
OG: Overground
10MWT: 10-Meter walk test
6MWT: 6-Minute walk test
sMERs: Spinal motor evoked responses
P: Paretic
NP: Non-paretic
EMG: Electromyography
RF: Rectus femoris
VL: Vastus lateralis
MH: Medial hamstring
TA: Tibialis anterior
MG: Medial gastrocnemius
VAF: Variability accounted for
RMT: Resting motor threshold
CI: Confidence interval
