# Supplementary material for: Noninvasive spinal stimulation improves walking in chronic stroke survivors: a proof-of-concept case series
Source: Biomed Eng Online. 2024 Apr 1;23:38. doi: 10.1186/s12938-024-01231-1 (PMC10986021; doi:10.1186/s12938-024-01231-1)
Supplement: Supplementary file 1 — Additional file 1: Table S1. Resting motor threshold (RMT, mA) of sMERs of each participant at each assessment and their Pre to Post changes. RMT resting motor threshold, sMER spinally motor evoked responses, TA tibialis anterior, MG medial gastrocnemius. [file 12938_2024_1231_MOESM1_ESM.docx]

**ADDITIONAL FILE**

**Noninvasive Spinal Stimulation Improves Walking in Chronic Stroke Survivors: A Proof-of-Concept Case Series**

**Authors:** Yaejin Moon^1,2,3,^ ^†^, PhD; Chen Yang^1,2,^ ^†^, PhD; Nicole C. Veit^1,4,^ ^†^, BS; Kelly A. McKenzie^1^, DPT; Jay Kim^1^, PhD; Shreya Aalla^1^, BS; Lindsey Yingling^1^, DPT; Kristine Buchler^1^, DPT; Jasmine Hunt^1^, BS; Sophia Jenz^2^, BS; Sung Yul Shin^1,2^, PhD; Ameen Kishta^1^, MS; V. Reggie Edgerton^5,6^, PhD; Yury P. Gerasimenko^7,8^, PhD; Elliot J. Roth^1,2^, MD; Richard L. Lieber^1,2,9^, PhD; Arun Jayaraman^1,2,*^, PT, PhD

^1^Shirley Ryan AbilityLab, Chicago, IL 60611, USA

^2^Feinberg School of Medicine, Northwestern University, Chicago, IL 60611, USA

^3^Department of Exercise Science, Syracuse University, Syracuse, NY 13057, USA

^4^Biomedical Engineering Department, McCormick School of Engineering, Northwestern University, Evanston, IL 60208, USA

^5^Rancho Los Amigos National Rehabilitation Center, Rancho Research Institute, Broccoli Impossible-to-Possible Lab, Downy, CA 90242, USA

^6^ Neurorestoration Center, Keck School of Medicine, University of Southern California, Los Angeles, CA 90033, USA

^7^ Kentucky Spinal Cord Injury Research Center, University of Louisville, Louisville, KY 40202, USA

^8^Pavlov Institute of Physiology, St. Petersburg, Russia

^9^Hines VA Medical Center, Maywood, IL 60141, USA

^†^Authors contributed equally to the manuscript

^*^**Corresponding Author**:

Arun Jayaraman, PT, PhD

Email: ajayaraman@sralab.org

**Contents:**

- - Additional Table 1. Resting motor threshold (RMT) of sMERs of each participant at each assessment and their Pre to Post changes

**Additional Table 1. Resting motor threshold (RMT, mA) of sMERs of each participant at each assessment and their Pre to Post changes.** RMT = resting motor threshold. sMER = spinally motor evoked responses. TA = tibialis anterior. MG = medial gastrocnemius.

| Participant | | TA RMT (mA) | | | | MG RMT (mA) | | | |
| --- | --- | --- | --- | --- | --- | --- | --- | --- | --- |
|  |  | Pre | Post | Raw change | % Change | Pre | Post | Raw change | % Change |
| Match 1 | Stim1 | 70 | 70 | 0 | 0% | 60 | 70 | 10 | 17% |
|  | Control1 | 155 | 145 | -10 | -6% | 140 | 135 | -5 | -4% |
| Match 2 | Stim2 | 170 | 130 | -40 | -24% | 195 | 110 | -85 | -44% |
|  | Control2 | N/A | N/A | N/A | N/A | N/A | N/A | N/A | N/A |
| Match 3 | Stim3 | 100 | 110 | 10 | 10% | 125 | 115 | -10 | -8% |
|  | Control3 | 105 | 95 | -10 | -10% | 70 | 65 | -5 | -7% |
| Match 4 | Stim4 | 145 | 120 | -25 | -17% | 165 | 130 | -35 | -21% |
|  | Control4 | 85 | 95 | 10 | 12% | 80 | 90 | 10 | 13% |
| **Mean**  **(SD)** | **Stim** | **121 (45)** | **108 (26)** | **-14 (23)** | **-8% (16)** | **136 (58)** | **106 (26)** | **-30 (41)** | **-14% (25)** |
|  | **Control** | **115 (36)** | **112 (29)** | **-3 (12)** | **-1% (12)** | **97 (38)** | **97 (35)** | **0 (9)** | **1% (11)** |
